# Genome-Wide Identification of Switchgrass Laccases Involved in Lignin Biosynthesis and Heavy-Metal Responses

**DOI:** 10.3390/ijms23126530

**Published:** 2022-06-10

**Authors:** Rui Li, Yan Zhao, Zhen Sun, Zhenying Wu, Honglun Wang, Chunxiang Fu, Hongbo Zhao, Feng He

**Affiliations:** 1 Shandong Provincial Key Laboratory of Energy Genetics, Qingdao Institute of Bioenergy and Bioprocess Technology, Chinese Academy of Sciences, Qingdao 266101, China; sunzhen@qibebt.ac.cn (Z.S.); wu_zy@qibebt.ac.cn (Z.W.); fucx@qibebt.ac.cn (C.F.); 2 College of Horticulture, South China Agricultural University, Guangzhou 510000, China; 3 College of Grassland, Resources and Environment, Inner Mongolia Agricultural University, Hohhot 010018, China; zhaoyannmg@imau.edu.cn; 4 Key Laboratory of Grassland Resources (IMAU), Ministry of Education, Hohhot 010018, China; 5 Key Laboratory of Biofuels, Qingdao Institute of Bioenergy and Bioprovess Technology, Chinese Academy of Sciences, Qingdao 266101, China; 6 Shandong Energy Institute, Qingdao 266101, China; 7 CAS Key Laboratory of Tibetan Medicine Research, Northwest Institute of Plateau Biology, Chinese Academy of Sciences, Xining 810008, China; hlwang@nwipb.cas.cn

**Keywords:** laccase, switchgrass, gene structure, lignification, heavy-metal stress

## Abstract

Plant laccase genes belong to a multigene family, play key roles in lignin polymerization, and participate in the resistance of plants to biotic and abiotic stresses. Switchgrass is an important resource for forage and bioenergy production, yet information about the switchgrass laccase gene family is scarce. Using bioinformatic approaches, a genome-wide analysis of the laccase multigene family in switchgrass was carried out in this study. In total, 49 laccase genes (*PvLac1* to *PvLac49*) were identified; these can be divided into five subclades, and 20 of them were identified as targets of miR397. The tandem and segmental duplication of laccase genes on Chr05 and Chr08 contributed to the expansion of the laccase family. The laccase proteins shared conserved signature sequences but displayed relatively low sequence similarity, indicating the potential functional diversity of switchgrass laccases. Switchgrass laccases exhibited distinct tissue/organ expression patterns, revealing that some laccases might be involved in the lignification process during stem development. All five of the laccase isoforms selected from different subclades responded to heavy metal. The immediate response of lignin-related laccases, as well as the delayed response of low-abundance laccases, to heavy-metal treatment shed light on the multiple roles of laccase isoforms in response to heavy-metal stress.

## 1. Introduction

Laccase (EC 1.10.3.2) belongs to the multicopper oxidase family, catalyzing the oxidation of a broad range of substrates using molecular oxygen as the final electron acceptor [1]. The catalytic center of laccase shares two conserved copper centers, including the T1 copper site and the T2/T3 trinuclear copper cluster. The reduction of T1 copper simultaneously couples the oxidation of a substrate and the reduction of dioxygen to water at the T2/T3 cluster. The oxidized substrate forms a free radical and undergoes nonenzymatic oxidation or reduction reactions [2]. This unique catalytic process grants laccases a nonspecific catalytic ability, allowing laccases to play multiple functions in living organisms, and to be utilized for biotechnological and environmental applications. Laccase is widely distributed in plants, fungi, bacteria, and insects. Although laccase was first discovered in the Japanese lacquer tree, the characterization and application of plant laccases is still limited compared with the research on microbe laccases [3]. Plant laccases belong to large, multigene families with high redundancy, which has restricted the understanding of their functions in vivo. In *Arabidopsis*, 17 laccase isoforms were annotated [4], while 24, 30, 52, and even 93 laccase sequences were identified in *Citrus sinensis* [5], *Oryza sativa* [6], *Setaria viridis* [7], and *Glycine max* [8]. Because of the functional redundancy of laccase genes, most of the laccase mutants identified in *Arabidopsis* exhibited no phenotype [4], and the genetic manipulation of a single laccase gene showed little influence on the phenotypes [7]. Consequently, recent approaches have proven that the comprehensive analysis of the laccase gene family in plants will accommodate the understanding of their properties and physiological functions. 

Lignin is a complex phenolic polymer deposited in the secondary cell walls of all vesicular plants. It consists of canonical hydroxycinnamyl alcohol monomers (known as monolignols) including *p*-coumaryl (H-type), coniferyl (G-type), and sinapyl (S-type) alcohols. Laccase and peroxidase are the main enzymes catalyzing the polymerization of monolignols. Recent studies have revealed that both the content and composition of lignin are influenced by laccases. The irregular xylem phenotype was observed in the stem of an *Arabidopsis lac4 lac17* double mutant with 40% less lignin content [9]. The phylogenetically related laccase genes in different plant species, including *Miscanthus sinensis* [10], *Brachypodium distachyon* [11], and *Populus tomentosa* [12], were also found to be involved in lignification. More recent research identified two laccase genes from Japanese cypress (*Chamaecyparis obtusa*) with different oxidation activities towards H-type and G-type monolignols, contributing to the precise localization of H-type and G-type lignin in distinct cell-wall layers [13]. Notably, a novel laccase (*ChLac8*) with caffeyl alcohol (C-type) oxidation activity was identified in *Cleome hassleriana*. The expression of *ChLac8* in the *Arabidopsis caffeic acid o-methyltransferase* mutant resulted in the formation of C-lignin in the stems of transgenic plants [14]. These recent advances further highlight the importance of laccases in lignin biosynthesis, demanding more genetic evidence to characterize the function of laccase in lignin polymerization.

Many heavy metals are considered essential for plant growth, but toxic to all life forms at high concentrations [15]. For example, copper (Cu) serves as a cofactor and as an activator of enzyme reactions in plants, and plays key roles in photosynthetic and respiratory electron transport [16]. However, excess Cu in the environment, caused by human activities, can be toxic to plants, inhibiting the growth of plants and impairing the photosynthesis process. As a barrier for plant defense against both abiotic and biotic stresses, lignin also plays an important role in the resistance of plants to excess heavy metals. It is speculated that the toxicity of Cu could be alleviated by promoting lignin biosynthesis [17]. Furthermore, excess Cu increased laccase activity in rice, and the expression of Cu-induced *OsLAC10* in *Arabidopsis* enhanced the tolerance of transgenic plants to Cu stress [6]. Plants also responded to other heavy metals including aluminum (Al), cadmium (Cd), and zinc (Zn), and accumulated lignin with different levels of sensitivity [18,19,20]. The process of lignin accumulation in the presence of heavy metals is correlated with induced laccase gene expression levels and enhanced activity of laccases in plant cell walls, but little is known about the functions of specific laccases in the process.

Switchgrass (*Panicum virgatum*) is a perennial grass that can be used for bioenergy and forage production [21]. In previous studies, we found that lignin modification through the manipulation of monolignol biosynthesis genes significantly enhanced the enzymatic saccharification yield of switchgrass biomass [22]. As a key player in monolignol polymerization, laccases provided promising targets for the genetic modification of the lignin biosynthesis pathway. Furthermore, it is indicated that switchgrass is also a promising platform resource for the bioremediation of contaminated soil. The overexpression of the switchgrass Cd-responsive gene *PvBip1* in *Arabidopsis* significantly improved the plant’s Cd tolerance [23]. Additionally, the engineered switchgrass expressing *xplA* and *xplB* efficiently removed hexahydro-1,3,5-trinitro-1,3,5-triazine (RDX) in a 3-year field trial [24]. 

Although laccase-directed lignification has been reported to be associated with the defense response against biotic and abiotic stresses, the function of switchgrass laccases in lignin biosynthesis, as well as their response to heavy-metal treatment, is still unknown. Thus, the aim of this study was to predict the *PvLac* candidates directly involved in lignification, and estimate their response to heavy metals in comparison with other laccase isoforms. In this study, we identified switchgrass laccase genes and analyzed their basic characteristics using bioinformatic methods. We then investigated the expression patterns of laccase candidates from each subclade upon heavy-metal treatment, and evaluated the laccase activity and lignin content in an excess copper environment. Our study highlighted the immediate response of lignin-related laccases, as well as the delayed response of low-abundance laccases to heavy-metal treatment, providing potential laccase candidates that respond to heavy-metal stress and regulate lignin biosynthesis.

## 2. Materials and Methods

### 2.1. Identification and Characterization of the Laccase Gene Family in Switchgrass

The laccase amino acid sequences from *Oryza sativa* [6] and *Arabidopsis*, obtained from the *Arabidopsis* information resource (TAIR, www.Arabidopsis.org/index.jsp, 5 October 2021), were used as queries for the BLAST (Basic Local Alignment Search Tool) searches against the switchgrass genome database in Phytozome v12.0 (*Panicum virgatum* v4.0, DOE-JGI, phytozome.jgi.doe.gov/, 6 October 2021). Using the HAMMER databases (www.ebi.ac.uk/Tools/hmmer/search/hmmscan, 10 October 2021), we further verified all candidate sequences according to the conserved plant laccase domains (Cu-oxidase: PF00394, Cu-oxidase_2: PF07731 and Cu-oxidase_3: PF07732). A total of 49 protein sequences were retrieved and numbered sequentially according to their position on the chromosomes. The chromosomal location information was download from Phytozome databases (phytozome-next.jgi.doe.gov/, 25 October 2021) and drawn using TBtools software [25]. Putative signal peptide length and cleavage site were predicted using SignalP (www.cbs.dtu.dk/services/SignalP/, 2 November 2021). The theoretical isoelectric point (pI) and molecular weight (MW) of the laccase proteins were calculated using the ExPASy website (web.expasy.org/compute_pi/, 2 November 2021).

### 2.2. Phylogenetic and Gene Duplication Analysis of the Laccase Proteins

The alignment of 17 AtLac, 29 OsLAC, and 49 PvLac peptides was performed using ClustalW in Mega 7.0 [26]. The phylogenetic tree was then constructed using MEGA 7.0 with the neighbor-joining (NJ) method (1000 bootstrap replicates), and then modified in iTOL (itol.embl.de/) [27]. Intra-species synteny blocks were analyzed using one-step MCScanX in TBtools [25]. The *Arabidopsis* and rice genome sequences and annotation information were downloaded from EnsemblPlants (http://plants.ensembl.org, 18 February 2022). Additionally, the collinearity between switchgrass and rice, and switchgrass and *Arabidopsis*, was analyzed and visualized using the one-step MScanX function in TBtools.

### 2.3. Gene Structure Analysis and Identification of Conserved Motifs as Well as Cis-Acting Elements

The exon–intron structures of *PvLac* genes were determined by comparing the coding sequence of each *PvLac* gene and its genomic sequence using Gene Structure Display Server (GSDS v2.0, http://gsds.cbi.pku.edu.cn/, 7 November 2021). The conserved domains of PvLACs were identified using the MEME server v5.4.1 (http://meme-suite.org/tools/meme, 7 November 2021). The promoter sequences of *PvLac* genes (2 kb upstream region) were retrieved from switchgrass genome database in Phytozome v12.0, and the *cis*-acting element analysis was performed using the online program PlantCARE (http://bioinformatics.psb.ugent.be/webtools/plantcare/html/, 17 November 2021). The miR397 target analysis of switchgrass laccases were conducted using psRNATarget (http://www.zhaolab.org/psRNATarget, 12 November 2021).

### 2.4. PvLac Expression Profiles in Various Switchgrass Tissues

The RNA-seq data on switchgrass laccase genes were obtained from JGI Plant Gene Atlas (JGI Plant Gene Atlas: An updateable transcriptome resource for improving structural annotations and functional descriptions across the plant kingdom, Sreedasyam et al., unpublished). The heatmap was constructed using the OmcStudio tools (https://www.omicstudio.cn, 17 December 2021). The expression data are shown in Table S1.

To elucidate the expression of *PvLacs* in different organs/tissues of switchgrass, the internodes, nodes, leaf blades, leaf sheaths, roots, and crowns were separately sampled with E4-stage switchgrass plants (growth stages determined using the criteria described by Moore et al. [28]. The relative expression level of selected *PvLacs*, as well as monolignol biosynthesis genes (*COMT* and *CCoAOMT*) in those samples, were then determined using qRT-PCR for collinear analysis.

### 2.5. Plant Materials and Treatments

The lowland switchgrass cultivar Alamo was used for all the experiments in this study. Seeds were spread on wet filter paper and incubated at room temperature for one week. The germinated seedlings were then transferred to 1/4 Murashige and Skoog (MS) solution to grow for another week. The growth solution was supplemented with different heavy-metal salts (CuSO_4_, NiCl_2_, FeSO_4_ and CdCl_2_) with concentrations of 100 μM and 500 μM for treatment. Samples were taken at 0 h, 6 h, and 24 h after treatment, immediately frozen in liquid nitrogen, and stored at −80 °C. E1-stage switchgrass plants were grown under the same conditions for 4 weeks, and different tissues of treated plants were collected for lignin analysis. Additionally, the seedlings were grown in 1/4 Murashige and Skoog (MS) under 4 °C for cold treatment, and 250 mM mannitol was supplemented for drought treatment. Samples were taken 1 h and 4 h after treatment.

### 2.6. RNA Extraction and qRT-PCR Analysis

Total RNA of samples was extracted using the Omega E.Z.N.A Plant RNA Kit (Omega Bio-Tek, Norcross, Georgia, USA) and reverse-transcribed into cDNA using Easyscript One-Step gDNA Removal and cDNA Synthesis SuperMix (TransGen Biotech Co., Ltd., Beijing, China). To quantify the relative expression level of laccases in response to heavy-metal stress, qRT-PCR was performed using 2 × SYBR Green qPCR Mix (Shandong Sparkjade Biotechnology Co., Ltd., Qingdao, China). *PvUbq2* (GenBank accession NO: HM209468) was used as reference to normalize the expression data. The primers used in this work are listed in Table S2. 

### 2.7. Protein Extraction and Enzyme Activity Assay

The switchgrass plant materials were ground well in liquid nitrogen and the soluble protein was extracted as described before [29]. Laccase activity was determined using 2, 2-azino-bis (3-ethylbenzothiazoline-6-sulfonic acid) (ABTS) as the substrate. The laccase activity was calculated as described by measuring the increase in absorbance at 420 nm [10].

### 2.8. Lignin Analysis 

Different tissues (leaf, root, and stem) of plants after heavy-metal treatment were harvested and oven-dried for 2 days at 55 °C. The dried samples were then ground using a CryoMill (Retsch GmbH, Haan, German) and the soluble component was removed via reflux extraction with acetone for 12 h. An acetyl bromide assay was performed to determine lignin content [22]. The cross sections of switchgrass internodes, as well as the root samples after heavy-metal treatment, were stained using the HCl-phloroglucinol method, as previously described [10]. 

## 3. Results

### 3.1. Identification and Characterization of LACs in the Switchgrass Genome

**Figure 1:**
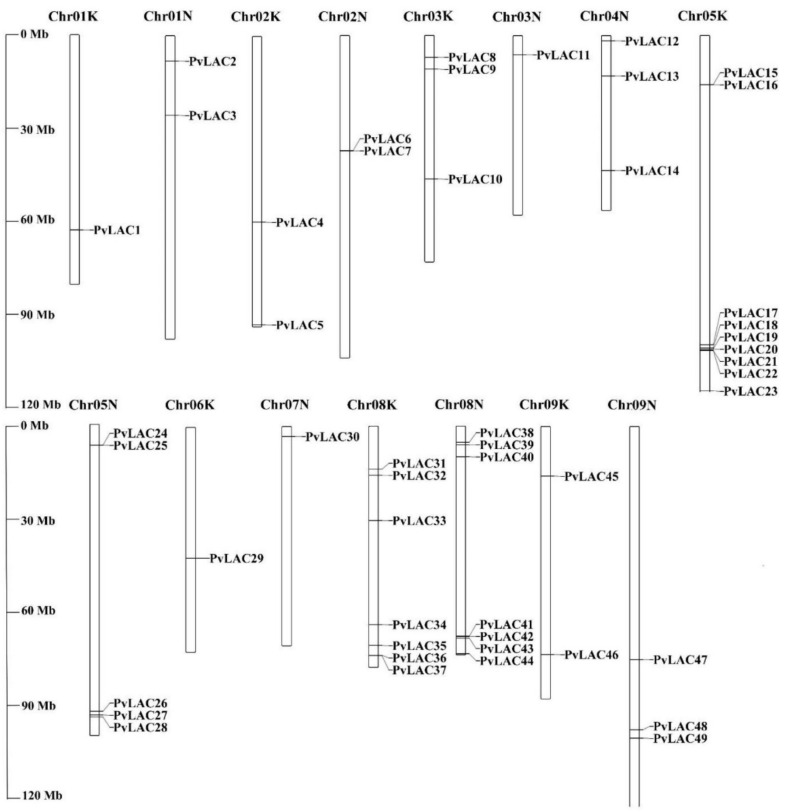
Chromosomal localization of switchgrass laccase gene family. The chromosomal localization of *PvLac* genes was based on the information provided in Phytozome v12.0. ChrK and ChrN are two sets of subgenomes of switchgrass (2n = 4x = 36). The scale bar is shown on the left.

To identify the laccase genes in the switchgrass genome, AtLAC and OsLAC amino acid sequences were used as queries to BLAST against the switchgrass genome database in Phytozome. The putative candidates were then verified according to the conserved plant laccase domains (PF07731, PF07732, and PF00394) (Figure S1) [30]. In total, 49 laccase gene sequences were identified and named *PvLac1* to *PvLac49*, according to their location on the chromosomes (Figure 1). The proteins encoded by the 49 identified *PvLac*s had 516 to 649 amino acid residues, and their molecular weights ranged from 57.95 kDa to 68.84 kDa. The theoretical isoelectric point (pI) analysis showed that the pI of switchgrass laccase proteins ranged from 5.19 (PvLAC10) to 9.78 (PvLAC14) (Table 1). More than 80% of switchgrass laccase had a signal peptide, which allows laccase to be secreted extracellularly (Table 1).

### 3.2. Phylogenetic Analysis of PvLac Family

**Figure 2:**
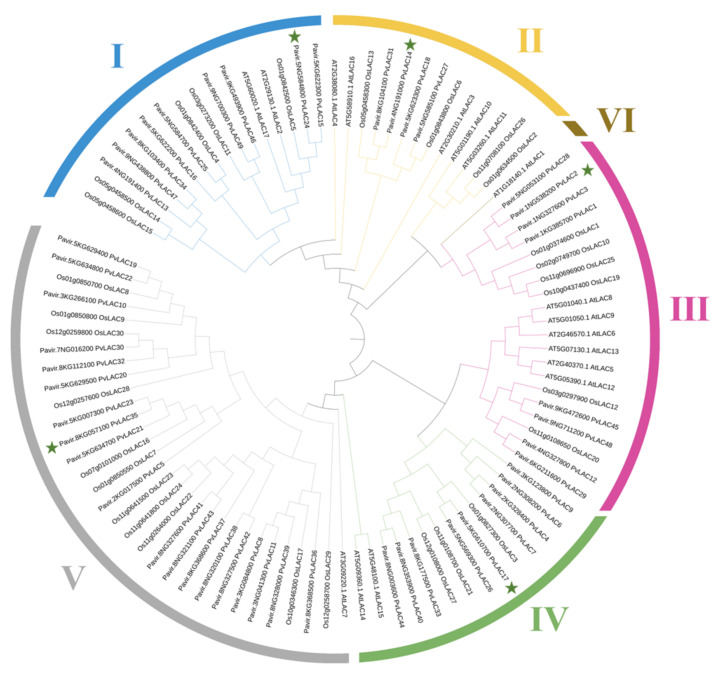
Phylogenetic analysis of PvLACs, OsLACs, and AtLACs. In total, 49 switchgrass, 29 rice, and 17 *Arabidopsis* laccase proteins were aligned using the ClustalW program, and the phylogenetic tree was constructed with MEGA 7.0 software using the neighbor-joining method with 1000 bootstrap replicates. The asterisks represent the five switchgrass genes selected from each subclade in this study.

To further understand the phylogenetic relationships among PvLAC proteins, we constructed a neighbor-joining (NJ) phylogenetic tree using 17 AtLAC, 29 OsLAC, and 49 PvLAC amino acid sequences. As shown in Figure 2, 49 switchgrass laccases can be divided into five subclades, based on the previously reported studies on *Arabidopsis* [31]; this indicates an evolutionary conservation between these two species. However, there are also distinct differences: switchgrass and rice laccase seem to lack the *Arabidopsis* homologous gene in subclade VI, while the members in the subclade V are largely expanded (11 OsLACs and 22 PvLACs). In detail, nine PvLACs are clustered in subclade I with AtLAC17, and four PvLACs are clustered in subclade II with AtLAC4. Both AtLAC17 and AtLAC4 were reported to be necessary for lignin biosynthesis [9].

### 3.3. Chromosomal Locations and Duplication Events in PvLac Gene Family

**Figure 3:**
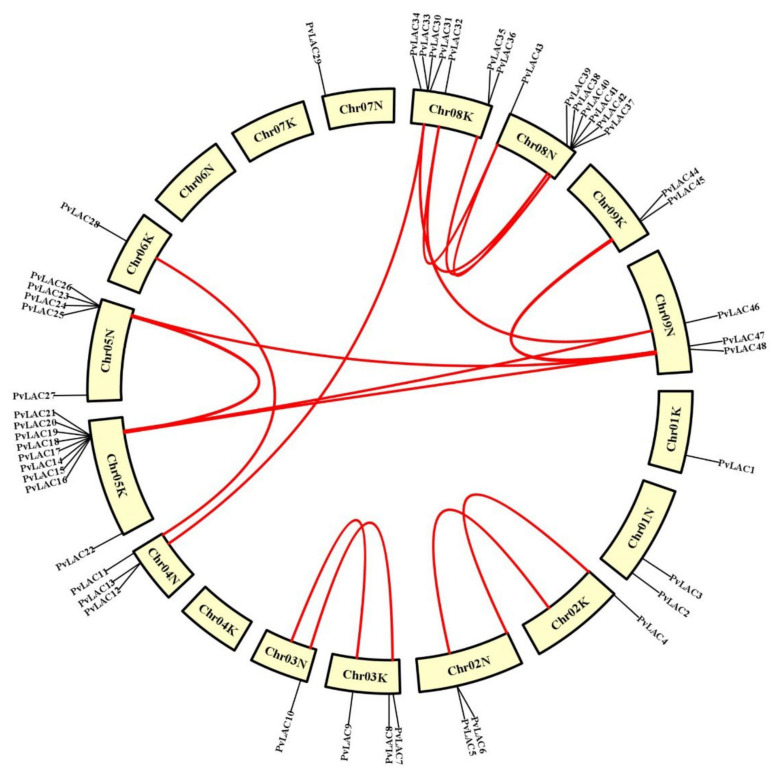
Colinear analysis of *PvLac* gene family. Red lines indicate duplicated *PvLac* gene pairs. The red line represented collinearity relationship between *PvLac* genes. Gene names are listed outside the circle, according to their chromosomal location.

Since switchgrass is an autotetraploid with two subgenomes (2n = 4x = 36), the localization of laccases on the chromosomes was analyzed. As shown in Figure 1, the laccase genes were unevenly distributed across 15 chromosomes. Most of the *PvLac* genes were found on Chromosome 05 K/N and Chromosome 08 K/N (14 on Chr05K/N and Chr08K/N, respectively), yet only one laccase gene was identified on Chr06K and Chr07N. Three *PvLac* genes were localized on Chr01K/N and Chr04K/N, while four were found on Chr02K/N and Chr03K/N. The *PvLac* genes were gathered irregularly on each chromosome; they localized next to each other close to the two ends of Chr05K/N and Chr08K/N due to tandem duplication (Figure 1, Table S2), while the distribution was relatively uniform on other chromosomes. Segmental and tandem duplication play important roles in the evolution of a large gene family. In total, three paralogous gene pairs of *PvLac* genes, in tandem duplication, were found in the genome (Figure 3, Table S3). More than 19 *PvLac* gene pairs were confirmed as segmental duplications localizing on different chromosomes, including *PvLac4*/*PvLac7*, *PvLac8*/*PvLac11*, *PvLac16*/*PvLac25*, *PvLac33*/*PvLac40,* and *PvLac45*/*PvLac48* on Chr02K/Chr02N, Chr03K/Chr03N, Chr05K/Chr05N, Chr08K/Chr08N, and Chr09K/Chr09N, respectively. The synteny analysis of laccase genes between switchgrass and rice, and switchgrass and *Arabidopsis*, revealed a higher collinearity between the *PvLac* and *OsLac* genes. In detail, 30 pairs with collinearity were found between the 22 *PvLac* genes and 12 *OsLac* genes, while only 13 pairs of collinearity were observed between the 8 *PvLac* genes and 4 *AtLac* genes (Figure S2). 

### 3.4. Gene Structure, miRNA Target Site Prediction and Conserved Motif Analysis of PvLac Family

**Figure 4:**
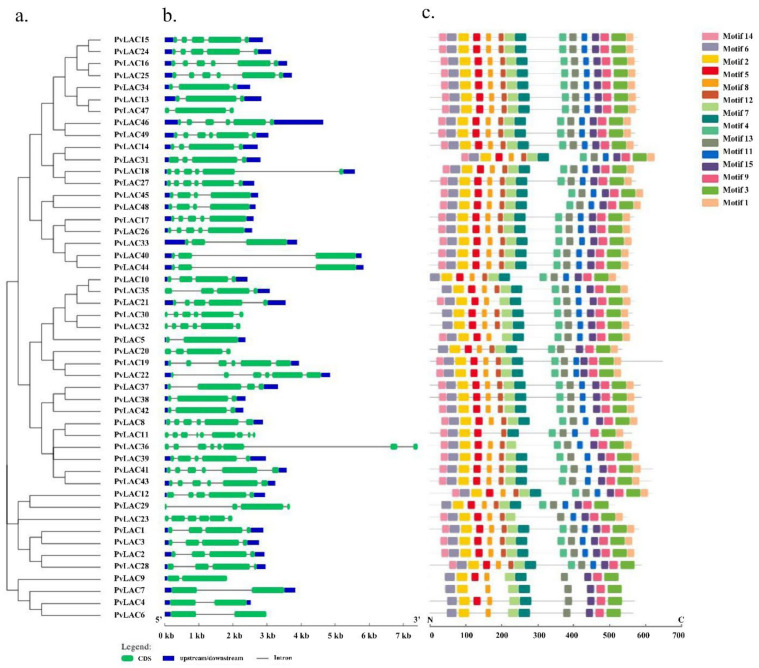
Structural analysis of laccases from switchgrass: (**a**) A neighbor-joining tree of 49 PvLACs constructed using MEGA 7.0; (**b**) the structure of 49 *PvLAC* genes. Green squares represent exons (coding sequences, CDS) and gray lines indicate introns, while the blue squares refer to the 5’ untranslated region (UTR) and 3’ UTR sequences; (**c**) conserved motifs of laccase genes in 49 switchgrasses. The colored square represents 15 conserved motifs, shown in supplementary table (Table S4).

Another NJ phylogenetic tree was constructed to align the 49 *PvLac* genes (Figure 4). *PvLac* genes with a closer genetic relationship tend to share a similar gene structure. The exon number of *PvLacs* varied from two to nine. *PvLac36* and *PvLac11* contained eight and nine exons, respectively, while *PvLac4*, *PvLac 5*, *PvLac 6*, *PvLac 7*, and *PvLac 9* exhibited only two. Most of *PvLacs* (36/49) contained three to five introns, among which long introns with a length greater than 2000 bp were found within *PvLac18*, *PvLac36*, *PvLac40*, and *PvLac44*.

To further elucidate the structure and function divergence of PvLAC proteins, we annotated 15 conserved motifs predicted by MEME on the full-length PvLAC sequences (Table S4, Figure 4c). In general, PvLACs shared similar motif profiles, especially members of the same subclade. Members of subclades I, III, and IV contained all 15 motifs, except PvLAC23 and PvLAC29; this indicates a possible functional redundancy among those members. Some motifs (motifs 1, 2, 6, 8, 11, and 14) were highly conserved and distributed across all PvLAC sequences, suggesting a potentially important role in the function of laccases. In subclade V, which is the most expanded subclade, nine laccases (PvLAC4, PvLAC6, PvLAC7, PvLAC9, PvLAC10, PvLAC20, PvLAC30, PvLAC32, and PvLAC35) lack motif 3. The distinct structural difference within PvLACs in the expanded subgroup may indicate functional divergence among proteins. 

The expression of plant laccases was tightly regulated by miRNAs, including miR397, miR408, miR528, and miR857. The miRNA397 family in plants mainly targets the laccase genes involved in the lignin biosynthesis process, providing reliable evidence to predict the function of laccases. The sequences of the 49 *PvLacs* were analyzed for the presence of miRNA target sites, and 20 of them were identified as the targets of miR397; however, only 4 and 2 *PvLacs* can be targeted by miR408 and miR857, respectively (Table S5). The results revealed complex post-transcriptional regulation of *PvLacs* by miRNAs, indicating the functional diversity of laccases in the development of switchgrass. 

### 3.5. Cis-Elements Analysis of PvLac Promoters 

In order to investigate the regulation mechanism of laccase expression in the presence of abiotic and biotic stresses, the *cis*-acting elements of laccase promoters were predicted using PlantCARE. In the promoters, hormone-responsive, stress-responsive, flavonoid biosynthetic, circadian-control and light-response elements were identified (Table 2, Figure S3). To be more specific, the 49 *PvLac* promoters contain hormone-responsive elements related to (methyl jasmonate) MeJA (41/49), auxin (25/49), gibberellin (33/49), and abscisic acid (47/49). All 49 of the *PvLac* promoters contained light-response elements, and 10 of them exhibited circadian-rhythm-control elements, which indicates a potential role of laccases in cell growth. Only promoters of *PvLac3*, *PvLac11*, and *PvLac48* share the flavonoid biosynthetic element, pointing to the involvement of these laccases in flavonoid biosynthesis. In addition, there were also abiotic stress response elements found across the 49 promoters, including elements responsive to drought, low temperature, and wounds. Based on the *cis*-element analysis results, the expression levels of *PvLac2*, *PvLac14*, and *PvLac17* were evaluated after treatments under low temperature (4 °C) and high osmotic pressure (250 mM mannitol). The cold treatment induced the expression level of *PvLac17* slightly, while both *PvLac2* and *PvLac17* responded to drought treatment significantly (Figure S4). The various regulatory elements predicted on the promoters of *PvLacs* confirmed the functional divergence of the laccase family, both in the stress response and plant growth in switchgrass.

### 3.6. Expression Patterns of PvLac Genes in Different Tissues

**Figure 5:**
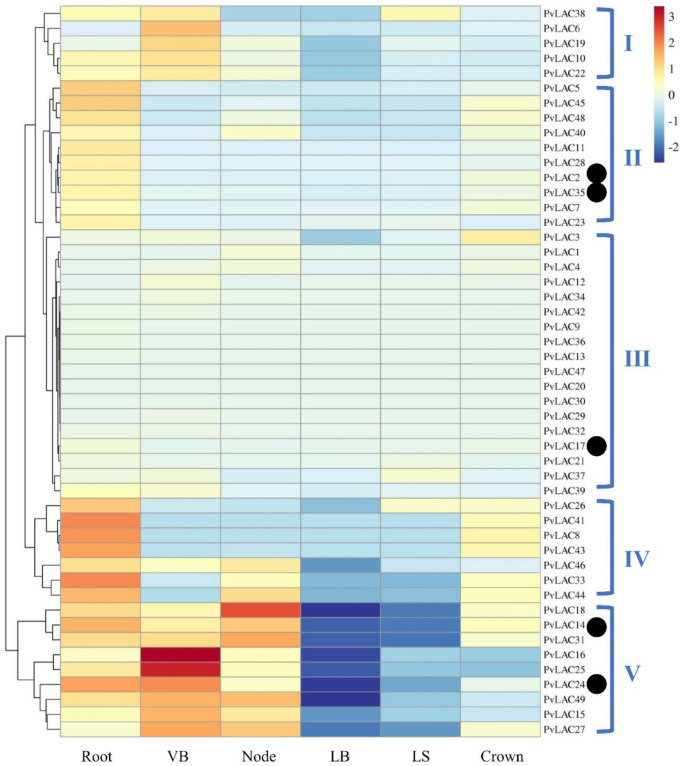
Expression profile of the *PvLacs* in E4 stage switchgrass. The detailed expression data were obtained from JGI Plant Gene Atlas (https://phytozome-next.jgi.doe.gov/geneatlas/, 9 December 2021) and are listed in Table S1. The selected ones in this study are marked with black dots. VB, vesicular bundle of the third internode; LB, leaf blade; LS, leaf sheath. The numbers listed in the right indicate the classification of *PvLacs* based on the tissue-specific expression data.

The expression pattern of plant laccases was tightly connected with their functions (He et al., 2019). To reveal the expression profiles of *PvLacs* in different tissues, a clustering heatmap was deduced based on the available expression data from Phytozome (https://phytozome-next.jgi.doe.gov/, 2021.12.09). Accordingly, the 49 switchgrass laccases can be classified into five subgroups according to their tissue-specific expression profiles (Table S1, Figure 5), suggesting various transcriptional regulation mechanisms for *PvLacs* along the switchgrass tissue. The members of subgroups IV and V showed the highest expression abundance among all the laccases, with an expression specificity in vascular bundles, nodes, and roots that were highly lignified. Conversely, the members of subgroup III were very poorly expressed. For example, *PvLac20*, *PvLac30*, and *PvLac47* were not active in any tissues. In addition, the expression of genes in subgroup II was restricted to roots and crowns with only trace expression in leaf blades and sheaths. The remaining laccase genes in subgroup I experienced a relatively mild expression profile, with moderate abundance in all the tissues except leaf blades.

To further evaluate the expression specificity of *PvLacs*, all the internodes, leaf sheathes, and leaf blades of E4-stage switchgrass plants were sampled for detailed expression analysis. Based on the heatmap, as well as the phylogenetic analysis, five genes from different subclades (*PvLac2*, *PvLac14*, *PvLac17*, *PvLac24,* and *PvLac35*) with high expression levels were selected (Figure 2) for expression analysis using qRT-PCR. Consistent with the mRNA-seq data, all selected laccases showed the highest expression abundance in internodes, except *PvLac17* (Figure S5a). Notably, *PvLac24* was expressed collinearly with two monolignol biosynthesis genes (*COMT* and *CCoAOMT*). The expression levels of *PvLac24* and *PvLac2* decreased along the stem and were positively correlated with lignin content (Figure S5b), potentially indicating a function in the lignification process.

### 3.7. Expression Patterns of Five PvLac Genes in Response to Various Heavy-Mental Treatments

**Figure 6:**
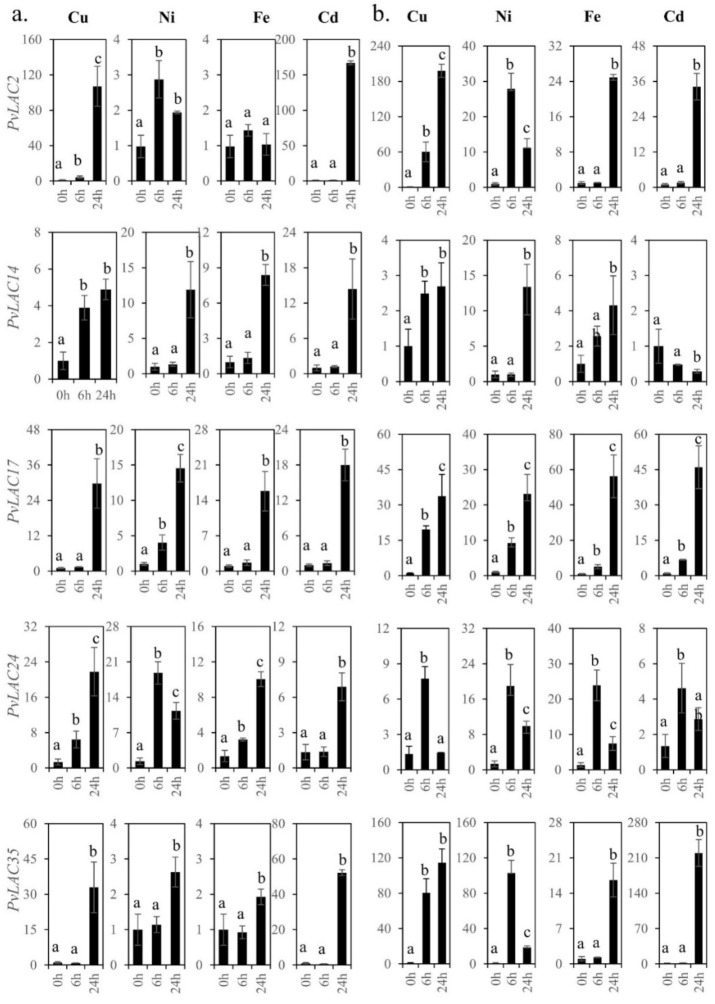
Response of *PvLac*s to heavy-metal treatment at concentrations of (**a**) 100 μM and (**b**) 500 μM. Significant differences are indicated by different letters (a, b, and c) after ANOVA analysis (*p* < 0.05). Error bars represent SD (n = 3).

Previous studies have shown that laccase genes responded to abiotic stresses and played a key role in plant defense (Song et al., 2018). The analysis of the *cis*-elements on *PvLac* promoters indicated that the *PvLac* genes may respond to multiple stresses. The expression level was induced for all the selected genes in response Cu, Ni, Fe, and Cd stress, either at a concentration of 100 μM or 500 μM, compared to the mock treatment control (Figure 6). Among the laccases selected, the expression levels of *PvLac2* and *PvLac35*, which were low in switchgrass (Figure 5 and Figure S5), were most significantly upregulated upon heavy-metal stress. After Cu and Cd treatment for 24 h, both genes experienced an over 100-fold induction of expression. All four of the heavy metals remarkably increased *PvLac17* expression level, even at a low concentration. Additionally, the two remaining laccases (*PvLac14* and *PvLac24*) were moderately induced by Cd and Cu, but appeared to be more sensitive to Ni. 

Under low-concentration treatment, the five laccases shared a similar induction pattern to Cd, while clear differences were observed in their response to Cu, Ni, and Fe (Figure 6a). Fe treatment did not alter the expression of *PvLac2*. However, only *PvLac14* and *PvLac24* expression levels were significantly upregulated after 6 h of Cu treatment at a 100 μM concentration. Conversely, most of the genes responded to heavy-metal stress quickly at 6 h, but underwent upregulation or downregulation at 24 h under high-concentration treatment (Figure 6b). In particular, the expression level of *PvLac24* significantly increased within 6h, followed by a sharp decrease at 24 h for all treatments at a 500 μM concentration.

To confirm the response of laccases to heavy-metal treatment, based on the RNAseq data (Table S1), two highly expressed laccase genes (*PvLac16* and *PvLac25*), as well as two laccases with no expression determined in any of the samples (*PvLac20* and *PvLac30*), were also selected for analysis. The expression levels of all the selected laccases were affected by heavy metals. However, the highly expressed laccases were only moderately upregulated, or even repressed, after heavy-metal treatment, while the laccases with low expression levels were significantly induced (Figure S4b).

### 3.8. Heavy-Metal Treatment Induced Both Lignin Content and Laccase Activity in Switchgrass

**Figure 7:**
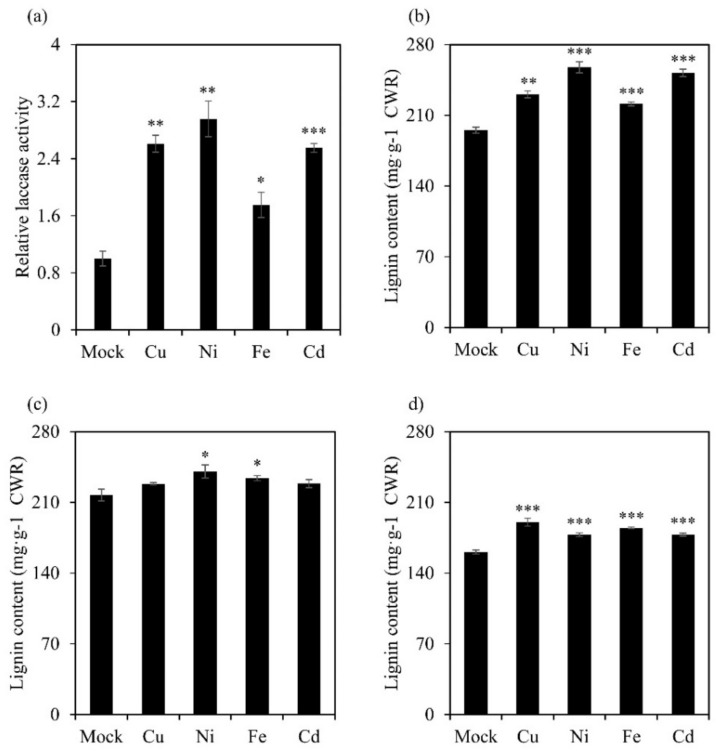
Lignin analysis and laccase activity analysis of switchgrass after heavy-metal treatments: (**a**) laccase activity of switchgrass after heavy-metal treatments. The E1-stage switchgrass was grown in 1/4 Murashige and Skoog (MS) solution supplemented with different heavy-metal salts (CuSO_4_, NiCl_2_, FeSO_4_, and CdCl_2_; 500 μM) for 4 weeks. Lignin content of switchgrass (**b**) root, (**c**) stem, and (**d**) leaf were measured after the treatment. Values are means ± SE (n = 3). The asterisks indicate significant differences calculated using t-test (*, *p* < 0.05; **, *p* < 0.01; ***, *p* < 0.001).

After a high concentration of heavy-metal treatment, the roots of switchgrass seedlings were stained red, suggesting an accumulation of lignin (Figure S6). The excess heavy metal also elevated the laccase activity in switchgrass. After treatment with Cu for one day, the laccase activity was induced 2.5-fold compared with the mock control. Nevertheless, treatment with Ni, Fe, and Cd also led to a 1.6- to 3-fold increase (Figure 7a), accompanied by the increased lignin staining.

To evaluate the impact of heavy-metal stress on lignin content and deposition, different samples (root, stem, and leaf) were collected for lignin analysis. The existence of heavy metals significantly induced the lignin content in the root and leaf. More specifically, excess Ni and Cd resulted in 32% and 29% more lignin in the roots, while the plant treated with Cu and Fe accumulated 18% and 15% more lignin in the leaves (Figure 7b,d). However, the degree of lignification in the stems of the treated plants was only slightly affected. Fe and Ni treatment increased the lignin content by 8% and 11%, respectively (Figure 7c).

## 4. Discussion

Laccases are ubiquitous copper oxidases widely distributed in fungi, bacteria, insects, and plants. The plant laccase multigene family is quite extensive, and because of the functional redundancy of plant laccase genes, the specific function of individual isoforms remains to be elucidated. Recent studies revealed that plant laccases were essential for secondary cell-wall biosynthesis through monolignol oxidation [10,13], pigment formation via flavonoid polymerization [32], cell morphology [9], and resistance to biotic and abiotic stresses [6]. Switchgrass is an important perennial crop that produces both forage and biofuel feedstock. However, knowledge about the function of switchgrass laccases is very limited. In this study, the laccase gene family in switchgrass was intensively studied, and the basic characteristics were investigated using bioinformatics. 

The laccase multigene family varies in isoform numbers among different plant species, with 17 in *Arabidopsis* [4], 23 in moso bamboo [33], 29 in sugarcane [34] and Brachypodium [11], 49 in poplar [35], and even 93 in soybean [36]. The genome of *Panicum virgatum* harbors an expanded laccase gene family with 49 members, which is comparable to the number observed in *Setaria viridis* [7]. The switchgrass laccases contained conserved copper-binding domains, and most of the putative proteins, including a secretion-signal peptide, were predicted to be localized extracellularly. However, the overall similarity among switchgrass laccase sequences was relatively low (Table S6), and the *PvLac* genes were different in structure, which indicates a functional diversity of laccases in switchgrass.

Similar to other monocot plants including rice [6] and maize [37], the switchgrass laccase family can be divided into five subclades, whereas dicot plants including the *Arabidopsis* [4] and citrus [5] laccase families had six subclades. Tandem and segmental duplication of laccases localized on Chr05 and Chr08 contributed to the expansion of laccase isoforms in switchgrass, leading to the expansion of subclade V laccase members. The absence of subclade VI members in grass genomes was reported, as well as the duplication of members in subclade V, specifically in species within the Paniceae tribe [6,8]. To elucidate the evolutionary significance of the loss and duplication of specific laccase subclade members, the functional characterization of individual members is required. However, the biological function of *Arabidopsis* subclade laccases (*AtLac1* and *AtLac6*) remains unknown. Recent studies revealed the neofunctionalization of grass subclade V laccase. The *Miscanthus* subclade V laccase *MsLac3* is mostly expressed in leaves. Stem-specific expression of *MsLac3* in the *Arabidopsis lac4 lac17* double mutant restored the lignin content, but failed to complement the growth phenotype [38]. The expression level of subclade V laccase *PtLac110* in poplar was significantly upregulated in tension wood, which suggests a potential role in “stress” lignin formation [39]. Additionally, a special subclade V laccase from *Cleome hassleriana*, ChLAC8, facilitated the polymerization of C-lignin both in vitro and in vivo [14]. It is very likely that the laccase genes in different subclades shared a redundant function in lignification based on the catalytic ability of the monolignols. The specialized functions of subclade V laccases indicate the importance of laccase in plant growth and development, suggesting the potential neofunctionalization of expanded subclade V laccase genes in switchgrass. 

To identify switchgrass laccases that potentially participated in lignification, a combination of phylogenetic analysis, tissue-specific expression pattern analysis, and collinear expression analysis with monolignol biosynthesis genes was carried out. It is a proven efficient way to predict laccase function via the clustering of laccase genes to a given group related to lignification. Laccases identified as orthologues of AtLAC17 in Brachypodium (BdLAC5) and Miscanthus (MsLAC1) were confirmed to play an important role in lignification [10,11]. In our study, nine switchgrass laccases were clustered with *AtLac17*, and six of them (*PvLac16*, *PvLac17*, *PvLac24*, *PvLac25*, *PvLac27*, and *PvLac49*) were dominantly expressed in the vascular bundle of mature stems and clustered into the same subgroup. Among those laccase candidates, *PvLac24* was predicted to be the best target of miR397, with the highest expectation (Figure S5). All five of the selected laccases from each subclade showed the highest expression in switchgrass internodes, in line with typical lignin biosynthesis genes *COMT* and *CCoAOMT*; this suggests a potential role for these PvLACs in lignification. Among them, only *PvLac14* and *PvLac24* were expressed (Figure S5) exclusively in the stem, suggesting the involvement of *PvLac14* and *PvLac24* in stem developmental lignification. However, the spatial expression profile is not sufficient to envision a major role of laccase in lignification. In *Arabidopsis*, decreased lignin content was observed in the seed coat of the *lac15* mutant as well as the stem of the *lac4 lac17* double mutant [9,40]; conversely, the *lac2* mutant significantly accumulated lignin in roots [41]. Since the majority of *PvLacs* were highly expressed in either stems or roots containing a significant number of lignified sclerenchyma cells, the major role of individual laccases clustered in different subclades in lignification still requires further determination. Taken together, our results provide genetic evidence that strongly suggests the involvement of *PvLac24* in stem developmental lignification, but an in vivo study of gain or loss of function is still required to understand the specific function of individual laccase isoforms in switchgrass.

The analysis of all *PvLac* promoters indicated the presence of various *cis*-elements, including certain elements related to abiotic stress, such as TC-rich repeats (defense and stress responsiveness), MYB-binding sites (drought-inducibility), and LTR elements (low-temperature responsiveness). Light-response elements including G-box, GT1-motif, GATA-motif, ACE, Sp1, and 3-AF1-binding site were intensively distributed in all *PvLac* promoters, suggesting a role of PvLAC proteins in morphogenesis and development [5]. Despite a generally equal distribution of *cis*-elements on the promoters of five selected laccases, the MeJA response element was particularly abundant within a 100 bp region on *PvLac2* and *PvLac17* promoters. We further confirmed that laccase isoforms respond differently to environmental stresses, including low temperature and high osmotic pressure. The apparent differences in abundance and distribution of *cis*-regulatory elements on promoters of *PvLac* isoforms indicate the functional diversity of *PvLACs* in multiple physiological processes. 

In plants, the lignification process is related to heavy-metal absorption and transportation. As a major component of the cell wall, lignin contains multiple functional groups which bind heavy-metal ions and, thus, protect the plant cells by preventing the absorption of heavy metal into the cytoplasm [42]. The increased degree of lignification in roots, induced by excess Cd and Cu, may play an important role in inhibiting the transport heavy metals from root to shoot [6,43]. A high concentration of heavy metals affects plants’ photosynthesis by reducing the chlorophyll content (Pb) or altering the photosynthetic electron-transport chain (Cu) in leaves; thus, it is toxic to plants [44]. The accumulated lignin induced by heavy-metal stress in switchgrass roots and leaves may, in turn, improve tolerance to heavy-metal-stress conditions. The expression of laccases also responds to heavy metals, regulating plant growth and stress tolerance by affecting lignin biosynthesis [45]. Excess Cu and Cd significantly induced the expression of laccase genes, resulting in enhanced laccase activity, as well as increased lignin content [6,46], while the expression level of all the selected switchgrass laccases increased significantly after Cu and Cd treatment. 

Based on the phylogenetic analysis, the laccase with the highest expression level in each of the four subclades (*PvLac24* from subclade I, *PvLac14* from subclade II, *PvLac2* from subclade III, and *PvLac35* from subclade V), as well as *PvLac17* from subclade IV, which is predicted to be a target of miR408, were selected to investigate their responses to heavy-metal treatment. Upon Fe stress, *PvLac17* showed the strongest response, whereas its *Arabidopsis* orthologue *AtLac12* played an important role in plant Fe homeostasis [47]. The overexpression of subclade III Cu-responsive laccase gene *OsLac10* in *Arabidopsis* significantly promoted root elongation under Cu stress [6]. Nevertheless, the expression level of switchgrass subclade III laccase *PvLac2* was upregulated 100 to 200 times after Cu treatment in this study, indicating a potential function of *PvLac2* under Cu stress. Interestingly, although *PvLac24*, which was presumed to affect developmental lignification, responded rapidly after low concentration treatment, the induction fold was relatively limited. Switchgrass laccases exhibited various tissue expression specificities with distinct background expression levels; one hypothesis is that some of them are not involved in developmental lignification, but rather, function in lignin deposition under stress [7]. The genetic evidence in our study identified *PvLac24* as a promising candidate involved in the lignification process during stem development. The differential responses of *PvLac14* and *PvLac17* to abiotic stresses suggest the functional diversity of individual laccase genes in the family. Our study revealed a primary response of the laccases that facilitate developmental lignification to heavy-metal stress, as well as a delayed induction of low-expression-level laccases; this highlights the potential complementary roles of switchgrass laccases in lignification and heavy-metal-stress tolerance.

## Figures and Tables

**Table 1:** Characterization of putative switchgrass laccase proteins. ORF, open reading frame; pI, isoelectric point.

Gene Name	Gene ID	Number of Amino Acids (aa)	Molecular Weight (kDa)	Signal Peptide Cleavage Site	Signal Peptide Length	ORF Length	pI
*PvLAC1*	*Pavir.1KG385700*	581	63.6	VAA-TP	24	1746	6.76
*PvLAC2*	*Pavir.1NG538200*	581	64.0	VAA-SP	24	1746	6.39
*PvLAC3*	*Pavir.1NG327600*	575	63.0	AQA-LR	24	1728	6.62
*PvLAC4*	*Pavir.2KG328400*	570	62.4	ARA-AT	25	1713	6.95
*PvLAC5*	*Pavir.2KG017500*	569	62.1	ADA-AV	24	1710	8.55
*PvLAC6*	*Pavir.2NG308200*	566	62.4	ARA-AT	24	1701	6.81
*PvLAC7*	*Pavir.2NG307700*	568	62.4	ARA-AT	20	1706	8.84
*PvLAC8*	*Pavir.3KG084800*	619	68.5	TMA-LP	25	1860	7.09
*PvLAC9*	*Pavir.3KG123800*	560	61.2	AEA-KV	19	1683	6.51
*PvLAC10*	*Pavir.3KG266100*	530	58.2	/	/	1593	5.19
*PvLAC11*	*Pavir.3NG041300*	563	62.1	TMA-LP	22	1692	6.44
*PvLAC12*	*Pavir.4NG327800*	619	67.1	/	/	1860	8.98
*PvLAC13*	*Pavir.4NG191400*	585	63.0	AEA-ET	32	1758	6.92
*PvLAC14*	*Pavir.4NG191000*	579	62.6	AAA-RT	29	1740	9.78
*PvLAC15*	*Pavir.5KG622300*	578	62.9	AEG-AI	24	1737	7.64
*PvLAC16*	*Pavir.5KG622200*	582	63.3	VQG-IT	28	1749	8.83
*PvLAC17*	*Pavir.5KG610700*	569	62.7	AGA-EV	22	1710	5.90
*PvLAC18*	*Pavir.5KG623300*	580	63.4	AAG-DT	34	1743	9.05
*PvLAC19*	*Pavir.5KG629400*	649	67.6	ADA-AT	16	1950	5.81
*PvLAC20*	*Pavir.5KG629500*	535	58.0	/	/	1608	5.95
*PvLAC21*	*Pavir.5KG634700*	570	60.7	AHA-AT	18	1713	5.82
*PvLAC22*	*Pavir.5KG634800*	647	67.2	ADA-AT	16	1944	6.10
*PvLAC23*	*Pavir.5KG007300*	549	60.6	VSS-AE	22	1650	9.63
*PvLAC24*	*Pavir.5NG584800*	578	63.0	TEG-AI	24	1737	7.21
*PvLAC25*	*Pavir.5NG584700*	584	63.4	VQG-IT	29	1755	8.73
*PvLAC26*	*Pavir.5NG569300*	569	62.8	AGA-EV	22	1710	5.97
*PvLAC27*	*Pavir.5NG585100*	573	62.8	AAG-DT	27	1722	8.87
*PvLAC28*	*Pavir.5NG053100*	589	65.5	/	/	1770	6.60
*PvLAC29*	*Pavir.6KG211600*	516	55.8	/	/	1551	6.24
*PvLAC30*	*Pavir.7NG016200*	564	62.2	GHA-AT	18	1695	8.76
*PvLAC31*	*Pavir.8KG104100*	637	68.7	/	/	1914	9.77
*PvLAC32*	*Pavir.8KG112100*	567	62.3	GRA-AI	19	1704	9.05
*PvLAC33*	*Pavir.8KG177500*	573	61.9	VVA-KE	25	1722	6.43
*PvLAC34*	*Pavir.8KG103400*	583	62.8	AEA-ET	29	1752	7.67
*PvLAC35*	*Pavir.8KG057100*	563	61.5	/	/	1692	5.72
*PvLAC36*	*Pavir.8KG368500*	576	63.3	AMA-AV	28	1731	5.86
*PvLAC37*	*Pavir.8KG368600*	587	64.7	GEA-AV	22	1764	6.04
*PvLAC38*	*Pavir.8NG320100*	589	65.0	GEA-AV	24	1770	6.33
*PvLAC39*	*Pavir.8NG328000*	596	65.2	AAG-AV	28	1791	6.43
*PvLAC40*	*Pavir.8NG353900*	565	61.4	VVA-KE	22	1698	6.62
*PvLAC41*	*Pavir.8NG327600*	621	69.3	AVA-AS	24	1866	6.13
*PvLAC42*	*Pavir.8NG327500*	589	65.3	GEA-AV	24	1770	6.11
*PvLAC43*	*Pavir.8NG321100*	617	68.8	AVA-AS	23	1854	6.10
*PvLAC44*	*Pavir.8NG003600*	565	61.4	VVA-KE	22	1698	6.62
*PvLAC45*	*Pavir.9KG472600*	607	66.0	ALA-EE	28	1824	8.58
*PvLAC46*	*Pavir.9KG493900*	571	62.3	SHG-AT	22	1716	8.64
*PvLAC47*	*Pavir.9NG438800*	585	63.0	AEA-ET	32	1758	6.92
*PvLAC48*	*Pavir.9NG711200*	601	65.3	ALA-EE	26	1806	8.43
*PvLAC49*	*Pavir.9NG700300*	571	62.3	SHG-AT	22	1716	8.74

**Table 2:** The *cis*-element analysis of *PvLAC* promoters. MeJA, methyl jasmonate. The plus signs indicate the existence of a corresponding element. The detailed *cis*-element information is shown in Figure S3.

Gene Name	Hormones-Responsive					Stress-Responsive			Flavonoid Biosynthetic	Circadian Control	Light Response
	MeJA	Auxin	Abscisic Acid	Gibberellin	Salicylic Acid	Low Temperature	Stress and Defense	Drought-Inducibility			
*PvLAC1*	+	+	+			+					+
*PvLAC2*	+		+	+				+			+
*PvLAC3*		+	+				+	+	+		+
*PvLAC4*	+		+				+	+			+
*PvLAC5*	+	+	+	+	+	+	+	+		+	+
*PvLAC6*	+		+	+			+				+
*PvLAC7*	+	+	+	+	+	+		+			+
*PvLAC8*	+	+	+		+						+
*PvLAC9*	+	+	+	+			+			+	+
*PvLAC10*	+	+	+	+				+		+	+
*PvLAC11*	+		+				+	+	+		+
*PvLAC12*	+	+	+	+	+	+	+	+		+	+
*PvLAC13*	+	+	+	+		+	+	+			+
*PvLAC14*	+		+	+	+						+
*PvLAC15*	+		+	+	+	+		+			+
*PvLAC16*	+		+		+			+			+
*PvLAC17*	+	+	+	+	+	+		+			+
*PvLAC18*	+		+			+		+			+
*PvLAC19*	+	+	+	+	+	+				+	+
*PvLAC20*	+	+	+	+			+	+			+
*PvLAC21*	+		+	+				+			+
*PvLAC22*	+		+	+	+	+	+	+		+	+
*PvLAC23*	+		+					+		+	+
*PvLAC24*	+		+	+		+	+	+			+
*PvLAC25*	+		+	+	+			+			+
*PvLAC26*	+	+	+	+		+		+			+
*PvLAC27*		+	+	+		+		+			+
*PvLAC28*	+		+	+				+			+
*PvLAC29*	+		+	+				+			+
*PvLAC30*	+	+	+	+	+		+				+
*PvLAC31*	+		+	+	+		+	+			+
*PvLAC32*			+	+		+		+		+	+
*PvLAC33*		+		+	+	+		+			+
*PvLAC34*	+	+	+		+	+		+			+
*PvLAC35*	+	+	+	+	+	+		+		+	+
*PvLAC36*	+		+		+		+	+			+
*PvLAC37*	+	+		+	+	+		+			+
*PvLAC38*	+	+	+	+	+			+			+
*PvLAC39*	+		+		+			+			+
*PvLAC40*			+		+	+		+			+
*PvLAC41*	+	+	+					+		+	+
*PvLAC42*	+		+		+			+			+
*PvLAC43*	+	+	+	+	+						+
*PvLAC44*			+		+	+		+			+
*PvLAC45*	+		+	+				+			+
*PvLAC46*	+	+	+				+	+			+
*PvLAC47*		+	+	+		+	+	+			+
*PvLAC48*			+	+			+	+	+		+
*PvLAC49*	+	+	+	+			+				+

## Data Availability

All data supporting the findings of this study are available within the paper and within its supplementary materials published online.

## Supplementary Materials

The following supporting information can be downloaded at: https://www.mdpi.com/article/10.3390/ijms23126530/s1.

## Abbreviations

Lac	Laccases
COMT	Caffeic acid 3-O-methyltransferase
CCoAOMT	Caffeyl CoA 3-O-methyltransferase

## References

[1] Giardina P., Faraco V., Pezzella C., Piscitelli A., Vanhulle S., Sannia G. (2010). Laccases: A never-ending story. Cell Mol. Life Sci..

[2] Awasthi M., Jaiswal N., Singh S., Pandey V.P., Dwivedi U.N. (2015). Molecular docking and dynamics simulation analyses unraveling the differential enzymatic catalysis by plant and fungal laccases with respect to lignin biosynthesis and degradation. J. Biomol. Struct. Dyn..

[3] Janusz G., Pawlik A., Swiderska-Burek U., Polak J., Sulej J., Jarosz-Wilkolazka A., Paszczynski A. (2020). Laccase Properties, Physiological Functions, and Evolution. Int. J. Mol. Sci..

[4] Cai X., Davis E.J., Ballif J., Liang M., Bushman E., Haroldsen V., Torabinejad J., Wu Y. (2006). Mutant identification and characterization of the laccase gene family in *Arabidopsis*. J. Exp. Bot..

[5] Xu X., Zhou Y., Wang B., Ding L., Wang Y., Luo L., Zhang Y., Kong W. (2019). Genome-wide identification and characterization of laccase gene family in Citrus sinensis. Gene.

[6] Liu Q., Luo L., Wang X., Shen Z., Zheng L. (2017). Comprehensive Analysis of Rice Laccase Gene (OsLAC) Family and Ectopic Expression of OsLAC10 Enhances Tolerance to Copper Stress in *Arabidopsis*. Int. J. Mol. Sci..

[7] Simoes M.S., Carvalho G.G., Ferreira S.S., Hernandes-Lopes J., de Setta N., Cesarino I. (2020). Genome-wide characterization of the laccase gene family in Setaria viridis reveals members potentially involved in lignification. Planta.

[8] Wang J., Feng J., Jia W., Fan P., Bao H., Li S., Li Y. (2017). Genome-Wide Identification of Sorghum bicolor Laccases Reveals Potential Targets for Lignin Modification. Front. Plant Sci..

[9] Zhao Q., Nakashima J., Chen F., Yin Y., Fu C., Yun J., Shao H., Wang X., Wang Z.Y., Dixon R.A. (2013). Laccase is necessary and nonredundant with peroxidase for lignin polymerization during vascular development in *Arabidopsis*. Plant Cell.

[10] He F., Machemer-Noonan K., Golfier P., Unda F., Dechert J., Zhang W., Hoffmann N., Samuels L., Mansfield S.D., Rausch T. (2019). The in vivo impact of MsLAC1, a Miscanthus laccase isoform, on lignification and lignin composition contrasts with its in vitro substrate preference. BMC Plant Biol..

[11] Le Bris P., Wang Y., Barbereau C., Antelme S., Cezard L., Legee F., D’Orlando A., Dalmais M., Bendahmane A., Schuetz M. (2019). Inactivation of LACCASE8 and LACCASE5 genes in Brachypodium distachyon leads to severe decrease in lignin content and high increase in saccharification yield without impacting plant integrity. Biotechnol. Biofuels.

[12] Qin S., Fan C., Li X., Li Y., Hu J., Li C., Luo K. (2020). LACCASE14 is required for the deposition of guaiacyl lignin and affects cell wall digestibility in poplar. Biotechnol. Biofuels.

[13] Hiraide H., Tobimatsu Y., Yoshinaga A., Lam P.Y., Kobayashi M., Matsushita Y., Fukushima K., Takabe K. (2021). Localised laccase activity modulates distribution of lignin polymers in gymnosperm compression wood. New Phytol..

[14] Wang X., Zhuo C., Xiao X., Wang X., Docampo-Palacios M., Chen F., Dixon R.A. (2020). Substrate Specificity of LACCASE8 Facilitates Polymerization of Caffeyl Alcohol for C-Lignin Biosynthesis in the Seed Coat of Cleome hassleriana. Plant Cell.

[15] Goyal D., Yadav A., Prasad M., Singh T.B., Shrivastav P., Ali A., Dantu P.K., Mishra S., Naeem M., Ansari A.A., Gill S.S. (2020). Effect of Heavy Metals on Plant Growth: An Overview. Contaminants in Agriculture: Sources, Impacts and Management.

[16] Yruela I. (2009). Copper in plants: Acquisition, transport and interactions. Funct. Plant Biol..

[17] Su N., Ling F., Xing A., Zhao H., Zhu Y., Wang Y., Deng X., Wang C., Xu X., Hu Z. (2020). Lignin synthesis mediated by CCoAOMT enzymes is required for the tolerance against excess Cu in Oryza sativa. Environ. Exp. Bot..

[18] Kováčik J., Bačkor M. (2007). Phenylalanine Ammonia-Lyase and Phenolic Compounds in Chamomile Tolerance to Cadmium and Copper Excess. Water Air Soil Pollut..

[19] Tahara K., Norisada M., Hogetsu T., Kojima K. (2005). Aluminum tolerance and aluminum-induced deposition of callose and lignin in the root tips of *Melaleuca* and *Eucalyptus* species. J. For. Res.-Jpn..

[20] Van de Mortel J.E., Almar Villanueva L., Schat H., Kwekkeboom J., Coughlan S., Moerland P.D., Ver Loren Van Themaat E., Koornneef M., Aarts M.G.M. (2006). Large Expression Differences in Genes for Iron and Zinc Homeostasis, Stress Response, and Lignin Biosynthesis Distinguish Roots of *Arabidopsis thaliana* and the Related Metal Hyperaccumulator *Thlaspi caerulescens*. Plant Physiol..

[21] Fu C., Mielenz J.R., Xiao X., Ge Y., Hamilton C.Y., Rodriguez M., Chen F., Foston M., Ragauskas A., Bouton J. (2011). Genetic manipulation of lignin reduces recalcitrance and improves ethanol production from switchgrass. Proc. Natl. Acad. Sci. USA.

[22] Wu Z., Wang N., Hisano H., Cao Y., Wu F., Liu W., Bao Y., Wang Z.Y., Fu C. (2019). Simultaneous regulation of F5H in COMT-RNAi transgenic switchgrass alters effects of COMT suppression on syringyl lignin biosynthesis. Plant Biotechnol. J..

[23] Song G., Yuan S., Wen X., Xie Z., Lou L., Hu B., Cai Q., Xu B. (2018). Transcriptome analysis of Cd-treated switchgrass root revealed novel transcripts and the importance of HSF/HSP network in switchgrass Cd tolerance. Plant Cell Rep..

[24] Cary T.J., Rylott E.L., Zhang L., Routsong R.M., Palazzo A.J., Strand S.E., Bruce N.C. (2021). Field trial demonstrating phytoremediation of the military explosive RDX by XplA/XplB-expressing switchgrass. Nat. Biotechnol..

[25] Chen C., Chen H., Zhang Y., Thomas H.R., Frank M.H., He Y., Xia R. (2020). TBtools: An Integrative Toolkit Developed for Interactive Analyses of Big Biological Data. Mol. Plant.

[26] Kumar S., Stecher G., Tamura K. (2016). MEGA7: Molecular Evolutionary Genetics Analysis Version 7.0 for Bigger Datasets. Mol. Biol. Evol..

[27] Letunic I., Bork P. (2016). Interactive tree of life (iTOL) v3: An online tool for the display and annotation of phylogenetic and other trees. Nucleic Acids Res..

[28] Moore K.J., Moser L.E., Vogel K.P., Waller S.S., Johnson B.E., Pedersen J.F. (1991). Describing and quantifying growth stages of perennial forage grasses. Agron. J..

[29] Liu Q., Zheng L., He F., Zhao F., Shen Z., Zheng L. (2015). Transcriptional and physiological analyses identify a regulatory role for hydrogen peroxide in the lignin biosynthesis of copper-stressed rice roots. Plant Soil..

[30] Lu S., Wang J., Chitsaz F., Derbyshire M.K., Geer R.C., Gonzales N.R., Gwadz M., Hurwitz D.I., Marchler G.H., Song J.S. (2020). CDD/SPARCLE: The conserved domain database in 2020. Nucleic Acids Res.

[31] Turlapati P.V., Kim K.W., Davin L.B., Lewis N.G. (2011). The laccase multigene family in *Arabidopsis* thaliana: Towards addressing the mystery of their gene function(s). Planta.

[32] Wei J., Zhang X., Zhong R., Liu B., Zhang X., Fang F., Zhang Z., Pang X. (2021). Laccase-Mediated Flavonoid Polymerization Leads to the Pericarp Browning of Litchi Fruit. J. Agric. Food Chem..

[33] Li L., Yang K., Wang S., Lou Y., Zhu C., Gao Z. (2020). Genome-wide analysis of laccase genes in moso bamboo highlights PeLAC10 involved in lignin biosynthesis and in response to abiotic stresses. Plant Cell Rep..

[34] Zhang W., Lin J., Dong F., Ma Q., Wu S., Ma X., Fatima M., Jia H., Ming R. (2019). Genomic and Allelic Analyses of Laccase Genes in Sugarcane (*Saccharum spontaneum* L.). Trop. Plant Biol..

[35] Lu S., Li Q., Wei H., Chang M., Tunlaya-Anukit S., Kim H., Liu J., Song J., Sun Y., Yuan L. (2013). Ptr-miR397a is a negative regulator of laccase genes affecting lignin content in *Populus trichocarpa*. Proc. Natl. Acad. Sci. USA.

[36] Wang Q., Li G., Zheng K., Zhu X., Ma J., Wang D., Tang K., Feng X., Leng J., Yu H. (2019). The Soybean Laccase Gene Family: Evolution and Possible Roles in Plant Defense and Stem Strength Selection. Genes.

[37] Caparrós-Ruiz D., Fornalé S., Civardi L., Puigdomènech P., Rigau J. (2006). Isolation and characterisation of a family of laccases in maize. Plant Sci..

[38] He F. (2019). Exploring the Function of Laccases from Miscanthus Sinensis in Lignin Biosynthesis. Ph.D. Thesis.

[39] Ranocha P., Chabannes M., Chamayou S., Danoun S., Jauneau A., Boudet A.M., Goffner D. (2002). Laccase down-regulation causes alterations in phenolic metabolism and cell wall structure in poplar. Plant Physiol..

[40] Liang M., Davis E., Gardner D., Cai X., Wu Y. (2006). Involvement of AtLAC15 in lignin synthesis in seeds and in root elongation of *Arabidopsis*. Planta.

[41] Khandal H., Singh A.P., Chattopadhyay D. (2020). The MicroRNA397b -LACCASE2 Module Regulates Root Lignification under Water and Phosphate Deficiency. Plant Physiol..

[42] Guo X., Zhang S., Shan X. (2008). Adsorption of metal ions on lignin. J. Hazard. Mater..

[43] Ederli L., Reale L., Ferranti F., Pasqualini S. (2004). Responses induced by high concentration of cadmium in *Phragmites australis* roots. Physiol. Plantarum..

[44] Mustafa G., Komatsu S. (2016). Toxicity of heavy metals and metal-containing nanoparticles on plants. Biochim. Biophys. Acta (BBA) Proteins Proteom..

[45] Ghori N.H., Ghori T., Hayat M.Q., Imadi S.R., Gul A., Altay V., Ozturk M. (2019). Heavy metal stress and responses in plants. Int. J. Environ. Sci. Technol..

[46] Yang Y., Cheng L., Liu Z. (2007). Rapid effect of cadmium on lignin biosynthesis in soybean roots. Plant Sci..

[47] Bernal M., Kramer U. (2021). Involvement of *Arabidopsis* Multi-Copper Oxidase-Encoding LACCASE12 in Root-to-Shoot Iron Partitioning: A Novel Example of Copper-Iron Crosstalk. Front. Plant Sci..

