# Supplementary material for: Genome-Wide Identification of Switchgrass Laccases Involved in Lignin Biosynthesis and Heavy-Metal Responses
Source: Int J Mol Sci. 2022 Jun 10;23(12):6530. doi: 10.3390/ijms23126530 (PMC9224244; doi:10.3390/ijms23126530)
Supplement: Supplementary file 1 [file ijms-23-06530-s001.zip › ijms-1747563-supplementary.pptx]

## Slide 1
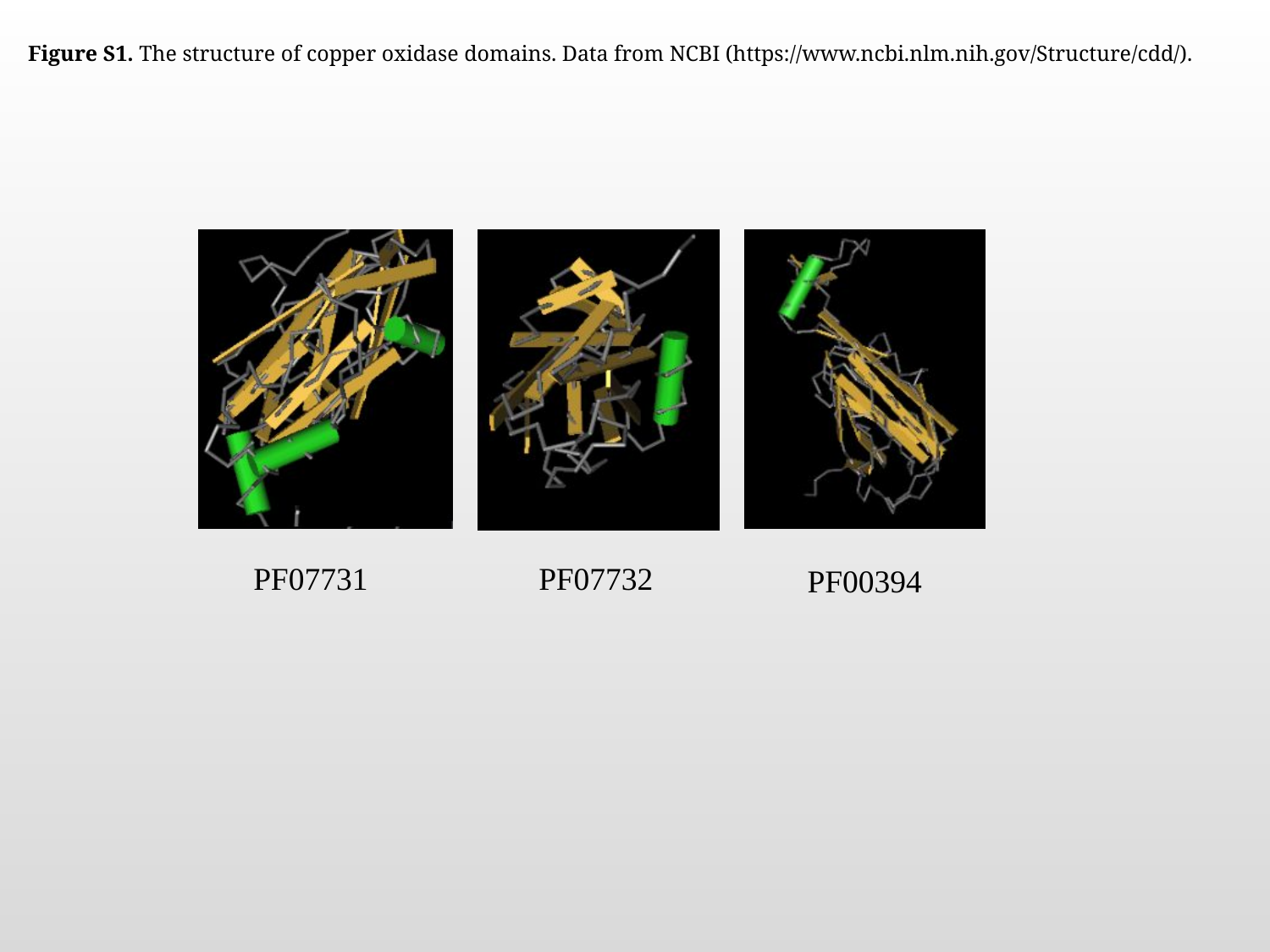

Figure S1. The structure of copper oxidase domains. Data from NCBI (https://www.ncbi.nlm.nih.gov/Structure/cdd/).
PF07731
PF07732
PF00394

## Slide 2
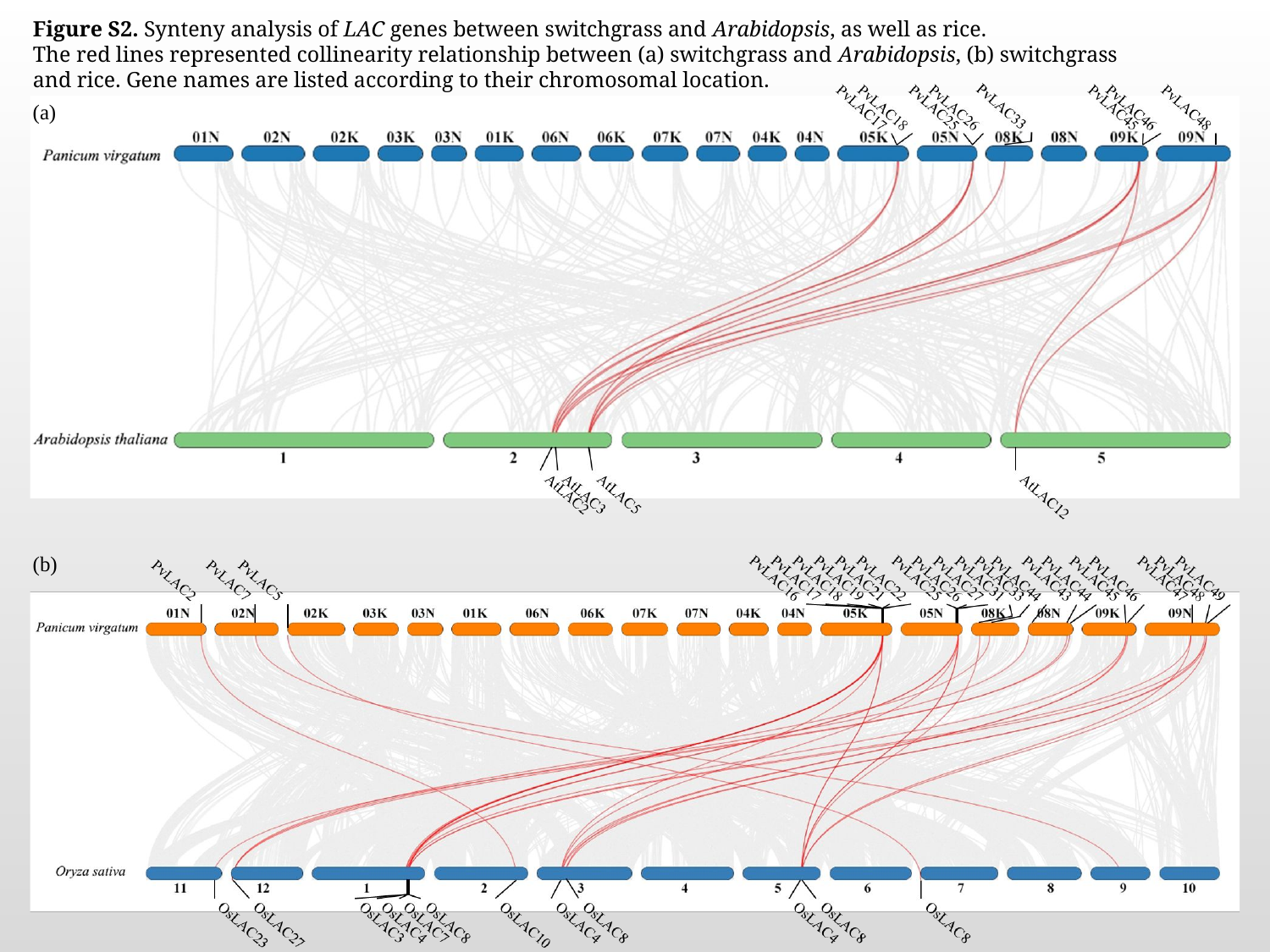

Figure S2. Synteny analysis of LAC genes between switchgrass and Arabidopsis, as well as rice.
The red lines represented collinearity relationship between (a) switchgrass and Arabidopsis, (b) switchgrass and rice. Gene names are listed according to their chromosomal location.
(a)
(b)

## Slide 3
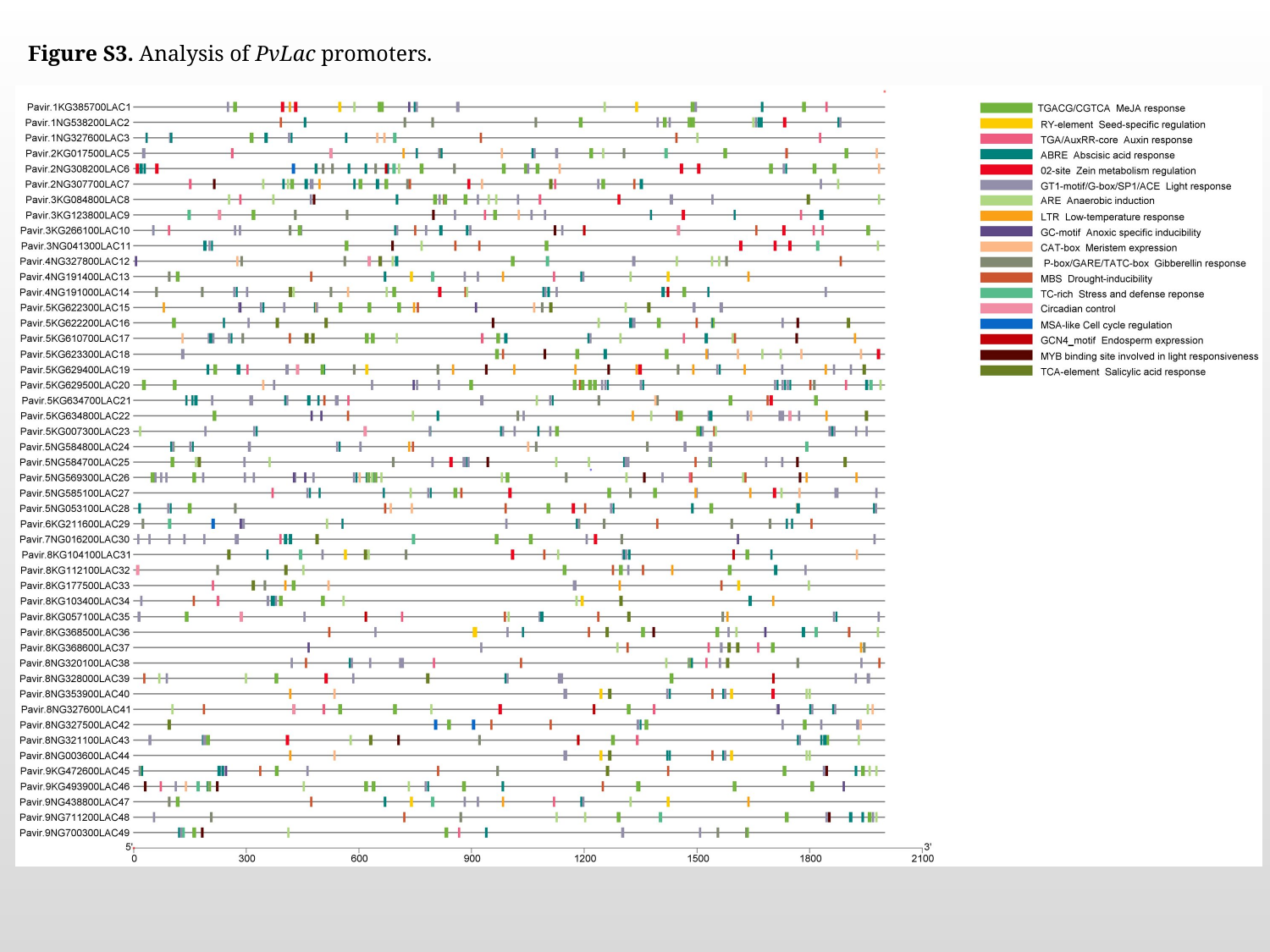

Figure S3. Analysis of PvLac promoters.

## Slide 4
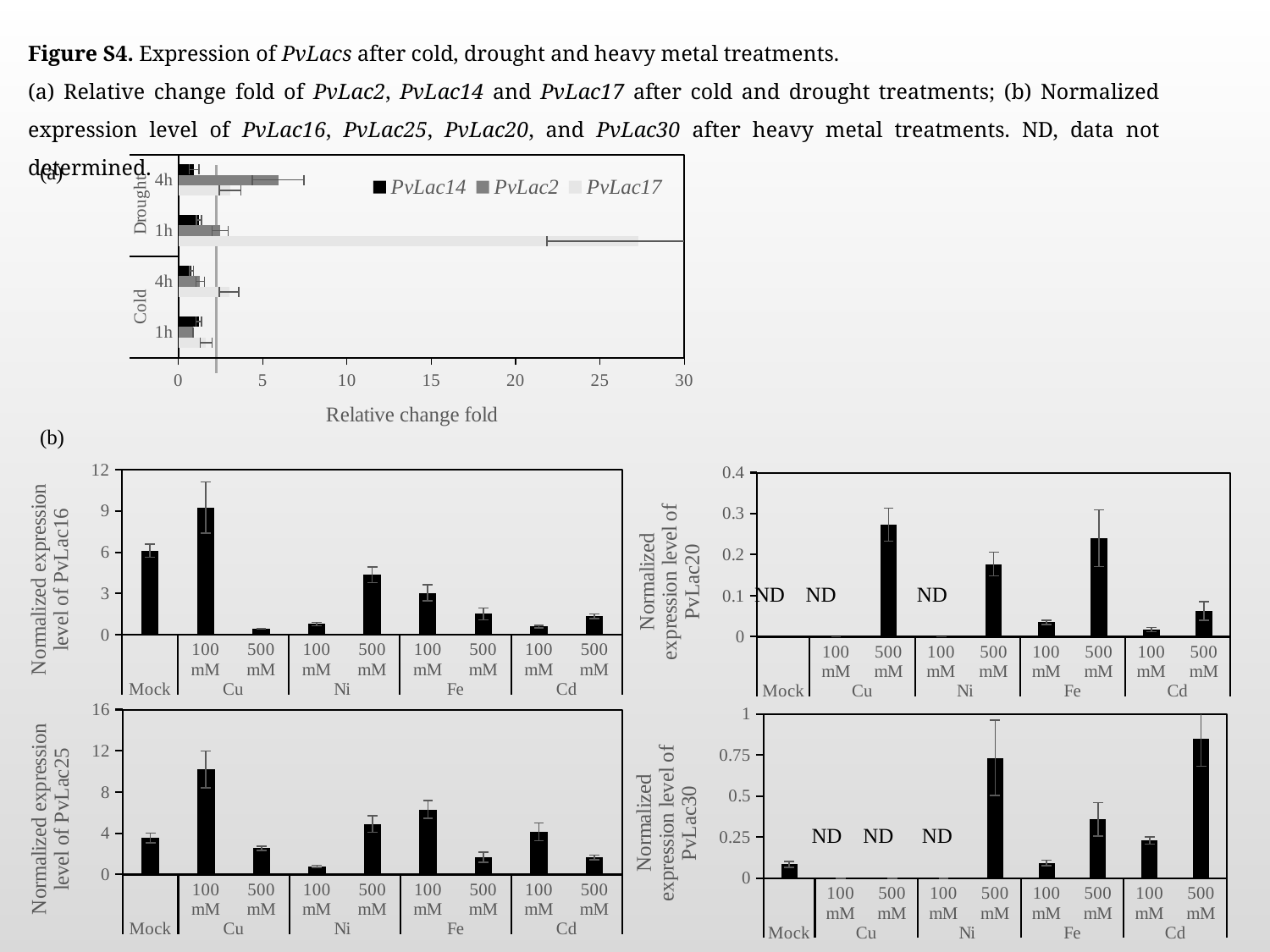

Figure S4. Expression of PvLacs after cold, drought and heavy metal treatments.
(a) Relative change fold of PvLac2, PvLac14 and PvLac17 after cold and drought treatments; (b) Normalized expression level of PvLac16, PvLac25, PvLac20, and PvLac30 after heavy metal treatments. ND, data not determined.
### Chart
| Category | PvLac17 | PvLac2 | PvLac14 |
|---|---|---|---|
| 1h | 1.6446878965283043 | 0.8650329643308913 | 1.2164259764276615 |
| 4h | 3.005887765817463 | 1.293614048791544 | 0.7828863009694977 |
| 1h | 27.273177235687378 | 2.4734323648575804 | 1.2275250404197426 |
| 4h | 3.0586698338213245 | 5.919980231436755 | 0.9315186880471519 |(a)
(b)
### Chart
| Category | LAC16 |
|---|---|
| | 6.114877094517066 |
| 100 mM | 9.244705812008359 |
| 500 mM | 0.44311834139364953 |
| 100 mM | 0.7850333493309096 |
| 500 mM | 4.363705493966974 |
| 100 mM | 3.050522500691025 |
| 500 mM | 1.5209831655454156 |
| 100 mM | 0.5989024490487683 |
| 500 mM | 1.3593733851827359 |
### Chart
| Category | LAC20 |
|---|---|
| | 0.0 |
| 100 mM | 0.0 |
| 500 mM | 0.27272142947360534 |
| 100 mM | 0.0 |
| 500 mM | 0.17712862193370632 |
| 100 mM | 0.03495378221528007 |
| 500 mM | 0.2399125545532537 |
| 100 mM | 0.01755750624274973 |
| 500 mM | 0.06281171491802505 |ND
ND
ND
### Chart
| Category | LAC25 |
|---|---|
| | 3.5594130233771852 |
| 100 mM | 10.184127044645816 |
| 500 mM | 2.5435239997189605 |
| 100 mM | 0.7925731388474789 |
| 500 mM | 4.898974573084355 |
| 100 mM | 6.3181878423776325 |
| 500 mM | 1.6886039069982575 |
| 100 mM | 4.151971562361508 |
| 500 mM | 1.6673649636880976 |
### Chart
| Category | LAC30 |
|---|---|
| | 0.08515607112207323 |
| 100 mM | 0.0 |
| 500 mM | 0.0 |
| 100 mM | 0.0 |
| 500 mM | 0.732332524982044 |
| 100 mM | 0.09419325259397916 |
| 500 mM | 0.3580906042231059 |
| 100 mM | 0.22980295608456502 |
| 500 mM | 0.8481714132541873 |ND
ND
ND

## Slide 5
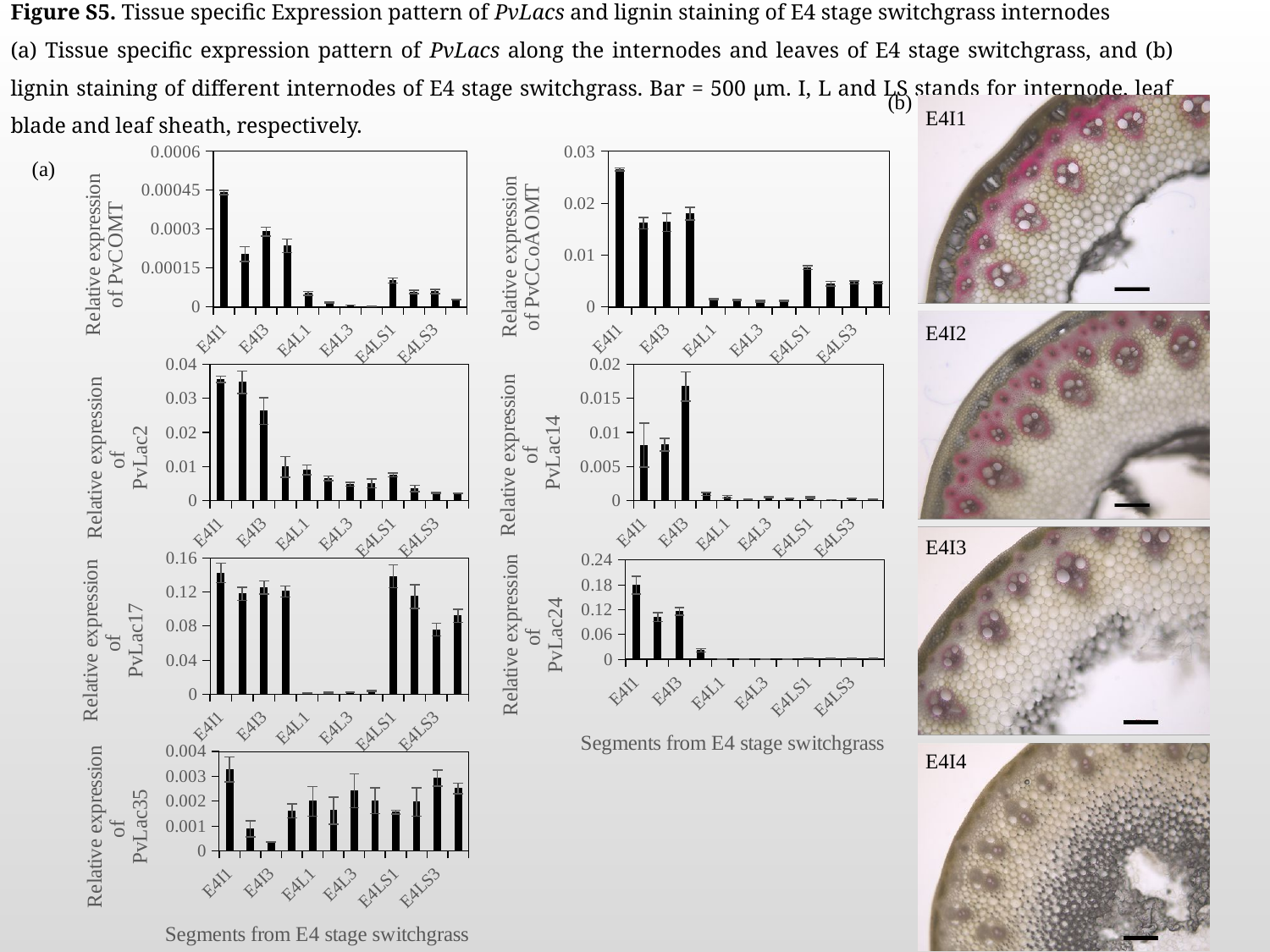

Figure S5. Tissue specific Expression pattern of PvLacs and lignin staining of E4 stage switchgrass internodes
(a) Tissue specific expression pattern of PvLacs along the internodes and leaves of E4 stage switchgrass, and (b) lignin staining of different internodes of E4 stage switchgrass. Bar = 500 μm. I, L and LS stands for internode, leaf blade and leaf sheath, respectively.
(b)
E4I1
### Chart
| Category | PvCOMT |
|---|---|
| E4I1 | 0.000441048748644886 |
| E4I2 | 0.000203584417791372 |
| E4I3 | 0.000291126727071444 |
| E4I4 | 0.000235862459390094 |
| E4L1 | 5.18036179114774e-05 |
| E4L2 | 1.63271325926924e-05 |
| E4L3 | 7.45585777697353e-06 |
| E4L4 | 3.42178116037824e-06 |
| E4LS1 | 0.000101612821559781 |
| E4LS2 | 5.77930958525347e-05 |
| E4LS3 | 5.95758857832571e-05 |
| E4LS4 | 2.86148301152132e-05 |
### Chart
| Category | PvCCoAOMT |
|---|---|
| E4I1 | 0.0265312153853052 |
| E4I2 | 0.0161866207258076 |
| E4I3 | 0.0163118679718746 |
| E4I4 | 0.0179564788118168 |
| E4L1 | 0.00151248359788527 |
| E4L2 | 0.0013787932825743 |
| E4L3 | 0.00111994919032313 |
| E4L4 | 0.00125059369701934 |
| E4LS1 | 0.0075948649865207 |
| E4LS2 | 0.00448509367385891 |
| E4LS3 | 0.00469650671313425 |
| E4LS4 | 0.00472186515992287 |
### Chart
| Category | PvLac2 |
|---|---|
| E4I1 | 0.0355197400880349 |
| E4I2 | 0.0346570188414676 |
| E4I3 | 0.0262231697874852 |
| E4I4 | 0.00989117541003566 |
| E4L1 | 0.00904313471661643 |
| E4L2 | 0.00655057205567576 |
| E4L3 | 0.00482786111791365 |
| E4L4 | 0.00506672702389297 |
| E4LS1 | 0.00753440016797934 |
| E4LS2 | 0.00358422841809381 |
| E4LS3 | 0.00218412590358454 |
| E4LS4 | 0.00219037226155171 |
### Chart
| Category | PvLac14 |
|---|---|
| E4I1 | 0.00812842134643534 |
| E4I2 | 0.0081931475655553 |
| E4I3 | 0.0167082865645953 |
| E4I4 | 0.000962617000562223 |
| E4L1 | 0.000567007512149354 |
| E4L2 | 0.000228756061518375 |
| E4L3 | 0.000538381556225987 |
| E4L4 | 0.000348170673945518 |
| E4LS1 | 0.000441277904186443 |
| E4LS2 | 0.000131920651395089 |
| E4LS3 | 0.000339108666055803 |
| E4LS4 | 0.000232900394823483 |
### Chart
| Category | PvLac17 |
|---|---|
| E4I1 | 0.142014185328482 |
| E4I2 | 0.117653132016471 |
| E4I3 | 0.125019174021551 |
| E4I4 | 0.120430812230492 |
| E4L1 | 0.00180102202147718 |
| E4L2 | 0.00187918776461636 |
| E4L3 | 0.00282759157168765 |
| E4L4 | 0.00403597271817302 |
| E4LS1 | 0.138067449732006 |
| E4LS2 | 0.114662092188775 |
| E4LS3 | 0.0756687855229078 |
| E4LS4 | 0.0917949091910237 |
### Chart
| Category | PvLac24 |
|---|---|
| E4I1 | 0.179270835720308 |
| E4I2 | 0.101956031031711 |
| E4I3 | 0.115429183271802 |
| E4I4 | 0.0216520671096064 |
| E4L1 | 4.4509695036132e-06 |
| E4L2 | 1.0712351905785e-05 |
| E4L3 | 1.16603301780612e-05 |
| E4L4 | 3.58079693099741e-05 |
| E4LS1 | 0.00230615870276961 |
| E4LS2 | 0.00152559952065873 |
| E4LS3 | 0.00147803015947356 |
| E4LS4 | 0.00204981965841775 |
### Chart
| Category | PvLac35 |
|---|---|
| E4I1 | 0.00327289505832851 |
| E4I2 | 0.000886893652135181 |
| E4I3 | 0.000355550968610028 |
| E4I4 | 0.00161103394698589 |
| E4L1 | 0.00199326849574909 |
| E4L2 | 0.00161952780198737 |
| E4L3 | 0.00241690949051236 |
| E4L4 | 0.00201316030336185 |
| E4LS1 | 0.00155140870523106 |
| E4LS2 | 0.00196285887441299 |
| E4LS3 | 0.00292942447023621 |
| E4LS4 | 0.0025037078508188 |(a)
E4I2
E4I3
E4I4

## Slide 6
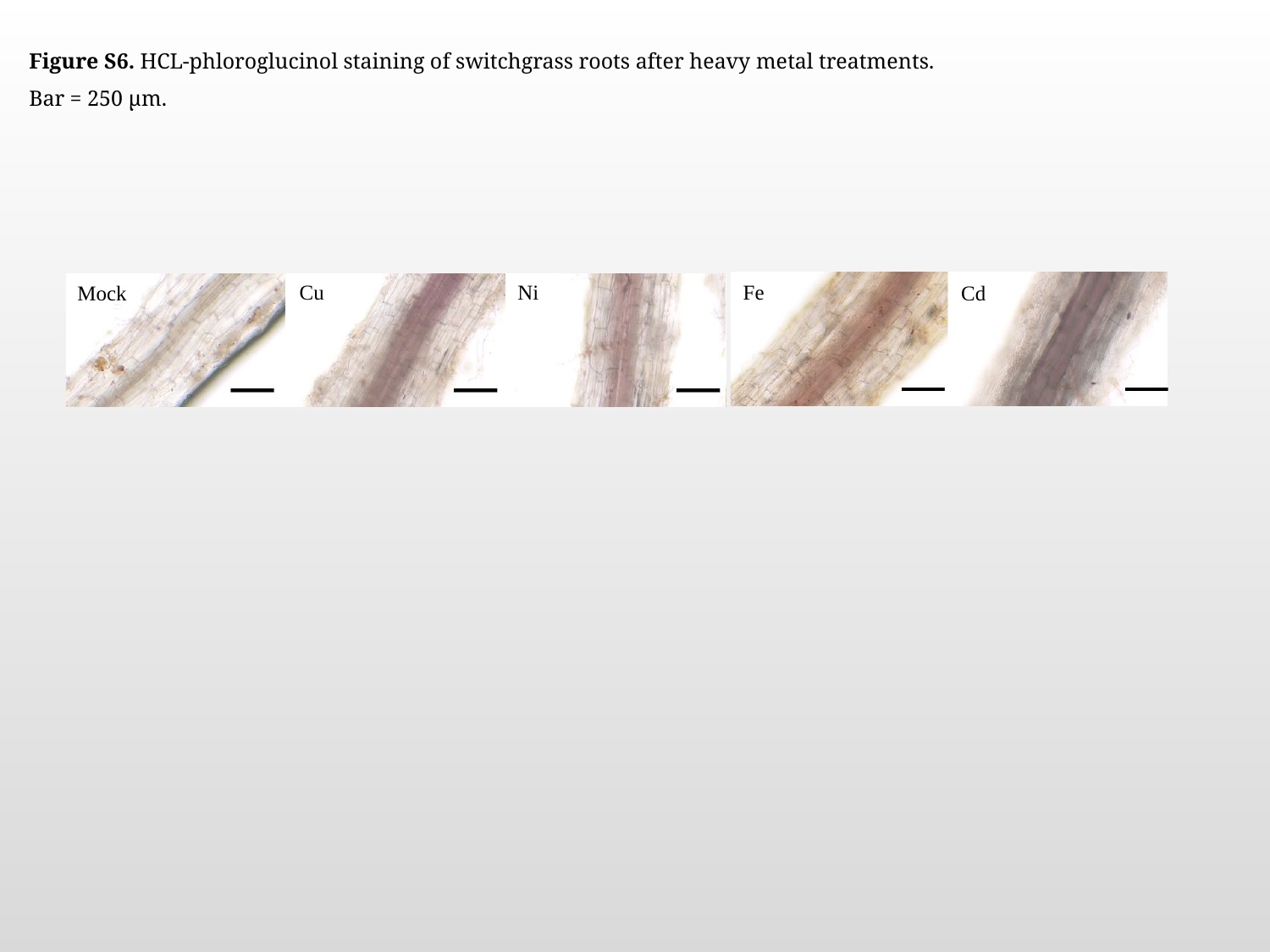

Figure S6. HCL-phloroglucinol staining of switchgrass roots after heavy metal treatments.
Bar = 250 μm.
Ni
Fe
Cu
Mock
Cd
